# Multimodal therapeutic intervention program associated with photobiomodulation therapy for individuals with chronic nonspecific neck pain: protocol for a clinical trial

**DOI:** 10.1186/s13063-024-08289-1

**Published:** 2024-07-03

**Authors:** Aron Charles Barbosa da Silva, Gabriela Nascimento Santana, Inaê Silva Santos, Patrícia Gabrielle dos Santos, Adriano Rodrigues de Oliveira, Almir Vieria Dibai Filho, Cid André Fidelis de Paula Gomes

**Affiliations:** 1 Postgraduate Program in Rehabilitation Sciences, University Nove de Julho, Vergueiro Street, N°: 235/249, Liberdade, São Paulo, SP CEP: 01504-001 Brazil; 2 Postgraduate Program in Physical Education, Federal University of Maranhão, São Luís, MA Brazil

**Keywords:** Neck pain, Low-level light therapy, Combined modality therapy, Exercise therapy, Rehabilitation

## Abstract

**Background:**

Neck pain is a prevalent global musculoskeletal issue, significantly contributing to the loss of years of healthy life due to disability. Chronic nonspecific neck pain (CNNP) involves diverse symptoms impacting mobility and quality of life. While therapeutic exercises demonstrate efficacy, the role of photobiomodulation therapy (PBMT) remains uncertain. This study aims to assess the additional effects of PBMT within a multimodal therapeutic intervention for CNNP.

**Methods:**

A randomized, two-arm, controlled, blind clinical trial follows CONSORT and SPIRIT guidelines. Participants diagnosed with CNNP will receive a stand-alone multimodal therapeutic intervention or the same program with additional PBMT. The primary outcomes will be assessed by the functional disability identified through applying the NDI (Neck Disability Index). Secondary outcomes will be pain intensity during rest and active neck movement, catastrophizing and kinesiophobia, functionality, and disability assessed at baseline, after 8 weeks, and at a 4-week follow-up. Both groups receive pain education before personalized interventions, including resistance exercises, neuromuscular activities, mobility, and body balance. The PBMT group undergoes low-level light therapy. Intention-to-treat analysis, using linear mixed models, employs data presented as mean, standard deviation, and differences with a 95% confidence interval. Non-normally distributed variables transform. Statistical significance is set at 5%.

**Discussion:**

This study addresses a critical gap in understanding the combined effects of PBMT and therapeutic exercises for CNNP. The findings aim to guide clinicians, researchers, and CNNP sufferers through rigorous methodology and diverse outcome assessments, offering valuable insights into evidence-based practices for CNNP management. Data confidentiality is maintained throughout, ensuring participant privacy during statistical analysis.

**Trial registration:**

Effects of adding photobiomodulation to a specific therapeutic exercise program for the treatment of individuals with chronic nonspecific neck pain, registration number: NCT05400473, on 2022-05-27.

**Supplementary Information:**

The online version contains supplementary material available at 10.1186/s13063-024-08289-1.

## Background

Globally, in 2020, it was estimated that around 494 million people had at least one musculoskeletal disorder [1]. Among these disorders, neck pain is one of the most prominent, with 22.1 million years of healthy life lost due to disability (YLDs) [2]. With a notable incidence in adult women and among individuals aged between 39 and 69 years, neck pain is responsible for 2.6 of YLDs of the global total [2].

Due to the vast magnitude of epidemiological reports related to neck pain. It is noted that it is a clinically diverse condition, marked by persistence and recurrence, most often characterized as chronic nonspecific neck pain (CNNP). Varying in intensity, location, and irradiation pattern among affected individuals, CNNP frequently results in functional limitations that impair quality of life, compromising mobility and the performance of daily living activities, in addition to emotional aspects, such as stress and anxiety, which contribute to the sensitization of the nervous system and the amplification of pain perception. In this way, all these components only confirm the considerable socioeconomic impacts of the CNNP on the loss of productivity, the increase in sick leave, and the financial costs of health care linked to its consequences [3–5].

All this complexity of signs, symptoms, and impairments is linked to CNNP. Several therapeutic approaches are suggested and tested in this scenario; for example, self-education interventions improve pain intensity, disability, kinesiophobia, and catastrophizing [6]. However, over the last few years, several studies have consolidated therapeutic exercises as a possible preventive intervention when the objective is to reduce the risk of an episode of neck pain. As the first line of treatment, with a frequency of at least three times a week, the aim is to improve function and pain in CNNP [7–10].

Even though no exercise is superior to others, there is significant variation in the methodological quality of the studies [11]. Therapeutic exercises involving resistance, mindfulness-based, and motor control are practical in promoting improvement in neck pain [12]. The frequency and duration of sessions are suggested to improve pain significantly during exercise, especially involving motor control [10]. Furthermore, when they include strengthening the cervical extensor muscles, they promote a reduction in neck pain and disability, using low and/or high loads, elastic resistance, and individualized therapeutic plans [13, 14].

In this context, several other resources have been tested for the consequences linked to CNNP. Over the last few years, photobiomodulation therapy (PBMT) has emerged and characterized as a light irradiation therapy that uses low-level laser therapy (LLLT) (light amplification by stimulated emission of radiation) and/or LEDs (light-emitting diodes) from the visible to the infrared spectrum, which, when interacting with tissues, promotes the release of growth factors related to epithelial cells, fibroblasts, and collagen proliferation. Furthermore, modulation of cellular behavior is characterized by the production of mitochondrial ATP and increased cellular metabolism, potential angiogenesis, and collagen synthesis. Physiological effects that potentially explain the clinical effects of using PBMT in tissue repair and reducing pain intensity [15].

Despite the cellular effects and consolidated mechanisms of action regarding using PBMT, the same does not occur when analyzing the results of the last five systematic reviews published between 2009 and 2022 involving its clinical use in CNNP [16, 17], which demonstrated that LLLT reduces pain up to 22 weeks after completion of treatment. The evidence does not support a safe conclusion about use [18–21].

In contrast, it indicates that using LLLT can benefit pain, function, and quality of life. Also, it suggests that the association of PBMT with therapeutic exercises seems to promote significant benefits only for pain intensity, highlighting that the combined use of these resources in individuals with CNNP does not seem to encourage a minimally effective clinical difference [19–22], and indicates that LLLT reduces neck pain, improves range of motion and pain pressure threshold, and can be used with other interventions such as manual therapy and therapeutic exercise.

In this context, the present study is justified, considering the complexity and high prevalence of CNNP, the lack of solidity in clinical findings related to the use of PBMT associated with therapeutic exercises, and the general recommendation of previously published systematic reviews, indicating the carrying out of new clinical trials following appropriate clinical guidelines and methodological processes. Therefore, the objective of this study will be to evaluate the additional effects of including PBMT in a multimodal therapeutic intervention program for individuals with CNNP. The structured hypothesis for the study is that the inclusion of PBMT in a multimodal therapeutic intervention program will improve functionality and disability, pain intensity, catastrophizing, kinesiophobia, and perception of the global effect of the treatment more than the use of a multimodal therapeutic intervention program alone in individuals with CNNP.

## Methods and design

### Design and setting

This will be a randomized, two-arm, controlled, and blind clinical trial. The research project’s methodology will be based on the methodological standards established by the CONSORT Statement [23]. In addition, the clinical trial protocol will follow the recommendations of the SPIRIT Statement (Standard Protocol Items: Recommendations for Interventional Trials (Table 1) [24]. The Template for Intervention Description and Replication (TIDieR) checklist will be used to describe and replicate interventions better [25].

**Table 1:**
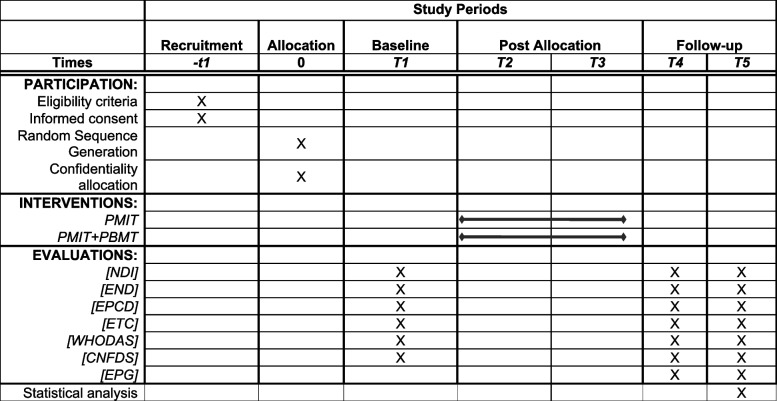
Schematic diagram of Spirit Statement overall study schedule

*−t1* time of recruitment of research participants, *T1* Evaluation 1, *T2* Phase 1 (4 weeks of intervention), *T3* Phase 2 (4 weeks of intervention), *T4* Evaluation 2, *T5* Evaluation 3, *PMIT*: Multimodal therapy Intervention Programme, *PMIT+PBMT* Multimodal therapy Intervention Programme+photobiomodulation therapy, *NDI* Neck Disability Index, *END* Numerical pain scale, *TAMPA* Kinesiophobia Tampa Scale, *EPC* Catastrophic Thoughts Scale

The research will be carried out in the movement laboratory at the University Nove de Julho, São Paulo, Brazil. It will be disseminated through posters, lectures in public health units, and consultations with the waiting list of rehabilitation clinics in São Paulo.

This study was approved by the Research Ethics Committee of University Nove de Julho nº 58616022.1.0000.5511 and was prospectively registered on the Clinical trials platform under number NTC05400473.

Participant recruitment began in July 2022 and is expected to continue until July 2025 (Table 1).

### Recruitment and participants

The sample calculation was processed using the Ene software, version 3.0 (Autonomous University of Barcelona, Spain). Therefore, functional disability measured using the Neck Disability Index (NDI) was chosen as the primary outcome variable. The calculation was based on detecting differences of 7 points between the groups, assuming a standard deviation of 7 [26, 27]. Thus, considering a statistical power of 80% and an alpha of 5%, a minimum number of 30 individuals per group was estimated.

Individuals with chronic nonspecific neck pain, defined as pain in the posterior cervical region between the superior nuchal line and the first thoracic spinous process and/or shoulder girdle, both genders, aged between 18 and 65 years, with neck pain for more than 90 days, NDI score ≥ 5, and Numerical Pain Scale (NPS) score ≥ 3 at rest or during active cervical movement [28, 29]. The exclusion criteria adopted in the present study will be neck pain associated with nerve root involvement (measured by clinical examination of dermatomes, myotomes, and reflexes), previous spine surgery, patients treated with physiotherapy for neck pain in the last 3 months to the study, spinal severe disorders such as fractures, tumors, inflammatory, and infectious diseases, any contraindication to low-power laser therapy, rheumatic, metabolic, neurological, or cardiopulmonary diseases, patients requiring artificial cardiac pacemakers, patients with deficits sensory, skin diseases, especially at the site of the current application, and history of tumors or cancer in the last 5 years [28, 29].

### Treatment allocation, randomization, and blinding

**Fig. 1:**
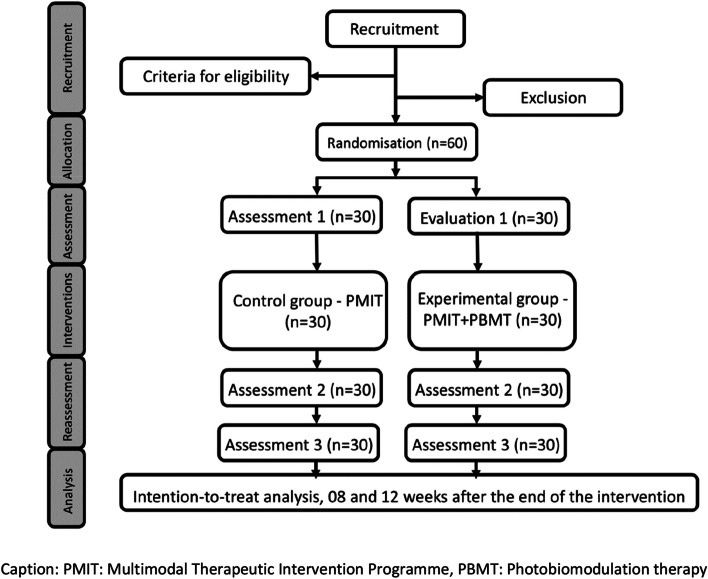
Flowchart of the study

The researcher responsible for the evaluations will need to determine which groups the participants will be allocated to. Therefore, one researcher will be responsible for recruitment and application of eligibility criteria and assessments, another for randomization and hidden allocation, and two researchers will be responsible for applying the treatment programs. At the same time, the last one will process and analyze the collected data. This way, the randomization process will be carried out through a random sequence in six blocks of 24 codes with an allocation rate 1:1, generated using software (Excel, Microsoft Corporation, Washington). The random codes will be written in opaque, sealed envelopes numbered sequentially from 1 to 60, thus ensuring the confidential allocation of research participants to study groups. After evaluating the eligibility steps, a researcher will open the envelopes and forward the research participants to the researcher responsible for their allocated group (Fig. 1). Research participants will be instructed to refrain from discussing the procedures during treatment with evaluators and other research participants.

### Outcome, mediators, and timing of measures

The primary outcome of the present study will be functional disability identified through the application of the NDI. In turn, the secondary outcomes will be the intensity of pain during rest and active movement of the neck, using the Numerical Pain Scale (NPS), catastrophizing, kinesiophobia, using the pain catastrophizing scale (PCS) and Tampa Scale for Kinesiophobia (TSK), functionality and disability using the World Health Organization Disability Assessment Schedule (WHODAS) and The Copenhagen Neck Functional Disability Scale (CNFDS), and perception of the global effect of the treatment using the Global Perception Scale (GPS).

Assessments will occur at three moments: after randomization, at the end of 8 weeks of the multimodal therapeutic intervention program, and after 4 weeks, at the end of the last session of the multimodal therapeutic intervention program.

### Neck Disability Index (NDI)

The NDI is a validated instrument for the Brazilian population, comprising ten questions investigating disability and neck pain. Responses are scored from 0 to 5, and the total score ranges from 0 to 50 points, based on the answers to assess the degree of disability due to neck pain. It has been previously validated and translated for the Brazilian population and reported a clinically important minimal difference of seven points out of the possible 50 for individuals with neck pain [26, 29, 30].

### Numerical Pain Scale (NPS)

The NPS is a simple scale from 0 to 10 to assess pain intensity, where 0 represents “no pain” and 10 describes the “worst pain imaginable.” The clinically significant difference was defined as 2.1 points. Pain intensity will be assessed based on the previous 7 days [31].

### Catastrophizing scale (PCS)

The PCS assesses pain-related catastrophizing and is an instrument adapted and validated for the Brazilian population. It consists of nine items scaled on a Likert scale ranging from 0 to 5 points associated with the words “almost never” to “almost always” at the endpoints. The total score is the sum of the items divided by the number of items answered, with a minimum score of 0 and a maximum of 5. There are no cutoff points, and higher scores indicate a more significant presence of catastrophic thoughts. A clinically important minimum difference of 20% of the total score was considered by the authors [32].

### Tampa Scale for Kinesiophobia (TSK)

This instrument will be used to assess fear of movement and fear of injury recurrence, validated for the Brazilian population [33]. It consists of 17 statements about pain, and the patient must indicate their level of agreement or disagreement with each statement using a 4-point scale. The final score can range from a minimum of 17 to 68 points. A higher score indicates a greater degree of kinesiophobia, suggesting that the individual is afraid to move due to neck pain. The authors considered a clinically important minimum difference to be 20% of the total score [33].

### World Health Organization Disability Assessment Schedule (WHODAS)

The WHODAS is a self-report assessment instrument developed by the World Health Organization (WHO) and designed to evaluate functionality and disability over the last 30 days. It has been translated, adapted, and validated with adequate measurement properties for Brazilian Portuguese. A Likert scale will be used for each item to define the severity of limitation, with a score of 0 denoting “no limitation” and 4 denoting “extreme limitation or inability to function.” The total score is the sum of all 12 items, where a score of 48 points represents the worst possible restriction. The clinically important minimum difference used as a reference will be 9 points [34–36].

### The Copenhagen Neck Functional Disability Scale (CNFDS)

The CNFDS is a unidimensional self-report scale comprising 15 items that assess the level of disability in daily activities in patients diagnosed with neck pain. When calculating the CNFDS score, the following procedures should be considered: For items 1 to 5, the response “yes” = 0, “sometimes” = 1, and “no” = 2; for items 6 to 15, the response “yes” = 2, “sometimes” = 1, and “no” = 0. Thus, the total score ranges from 0 to 30, with a higher score indicating greater disability [37, 38].

### Global Perception Scale (GPS)

The perception of the overall treatment effect by the research participant will be assessed using the Global Perception of Change Scale. The Global Perception of Change is a direct scale of the patient’s self-perception during the intervention. This scale consists of 11 points, ranging from −5 (worsening compared to the start of treatment), 0 (neutral), to +5 (improvement compared to the beginning of treatment), using the Portuguese version [39].

## Interventions

Participants will undergo a multimodal intervention program with two weekly sessions, totaling 16 sessions of approximately 50 min each over 8 weeks. The interventions will be conducted individually in reserved rooms, providing suitable lighting and climate conditions. Two researchers with experience in the clinical practice of chronic pain will administer previously standardized programs after a training period [40].

Before the intervention begins, all groups will receive a 45-min pain education session covering pathophysiological mechanisms, coping strategies, prevention of hypervigilance, deconstruction of beliefs and myths about chronic pain, and information about exams and alternative and surgical treatments. The educational session will employ expository, verbal, and visual approaches based on the visual content of the Retrain Pain Foundation [41].

### Multimodal Therapeutic Intervention Program

Comprising 30 participants, this group will undergo a multimodal therapeutic intervention program consisting of interventions for the cervical region, divided into phase 1 — with 11 interventions, and phase 2 — with 24 interventions, based on previous studies [42–44].

The multimodal therapeutic intervention program will include resistance exercises, neuromuscular activities, mobility, and body balance. Over 8 weeks, participants will have two individual sessions of the multimodal therapeutic intervention program, each lasting approximately 50 min. This totals 16 therapeutic intervention sessions, with a minimum of 24 h between sessions. Interventions will involve up to three sets of 8–12 repetitions or 10–15 s each, with rest intervals of 120 s between sets [44] (Additional file 1).

Interventions using elastic band resistance will be performed with 2-m bands featuring five resistance levels and therapeutic ball exercises of 50 and 60 cm. Throughout the multimodal therapeutic intervention program, the researcher will be close to the research participant, providing instructions and support and ensuring safety during activities [44, 45].

### Group 2—Multimodal therapeutic intervention program + photobiomodulation therapy

This group, consisting of 30 participants, will follow the same multimodal therapeutic intervention program as Group 1 (phases 1 and 2). However, photobiomodulation therapy will initially be applied to the cervical region.

The photobiomodulation therapy protocol will use a low-power infrared laser therapy unit. The unit has the following specifications: continuous optical output of 100 mW, a wavelength of 808 nm, beam size area of 0.028 cm^2^, power density of 1.78 W/cm^2^, delivering 7 Joules per point, and an application duration of 70 s for each point (Table 2).

**Table 2:** Description of PBM irradiation parameters

Equipment details	
Photobiomodulation (*Light Amplification by Stimulated Emission of Radiation)*	Infrared laser
Wavelength	808 nm (± 10 nm)
Manufacturer/type	DMC - Therapy ACP
Frequency	50–60 Hz
Operating mode	Continuous
Power output	100 mW (±20%)
Useful diameter of the optical fibers	1000 µm per fiber (0.01 cm^2^)
Power density	10 W/cm^2^
Energy	7 J (by application site)
Power density	700 J/cm^2^
Treatment time per point	70 s per point
Number of diodes	12 points (2 points in C2, 2 points in C3, 2 points in C4, 2 points in C5, 2 points in C6, 2 points in C7)
Total energy (dose)	84 J
Total treatment time	840 s
Application method	The applicator is in a stationary position, perpendicular to and resting against the muscles, 2 cm lateral to the spinous processes of C2 to C7. The volunteer will be sitting upright in a chair with dorsal support.

*nm* Nanometers, *Hz* Hertz, *nW* Nanowatts, *µm* Micrometer, *cm*^*2*^ Centimeters, *J* Joules, *s* Seconds, *C* Cervical spine

The research participant will be seated with the cervical region exposed for application. The researcher will position themselves posterior to the cervical spine, placing the tip of the laser therapy unit perpendicular to each of the 12 pre-defined points along the cervical region: 2 cm laterally to the spinous processes from C2 (second cervical vertebra) to C7 (seventh cervical vertebra), six points laterally to the right, and six points laterally to the left.

The interventions to be used are conservative interventions with low risks of events or side effects. However, researchers will check research participants for side effects related to therapeutic protocols weekly. If any event or side effect is recorded, the research team will assist the research participant, and the study will be terminated.

## Statistical analysis

Continuous and categorical variables will be described as mean ± SD (or median)) and frequency (%). After evaluating the normality distribution using the Kolmogorov-Smirnov test, parametric or non-parametric methods will be used. To check the quality of randomization and find potential confounders in the univariate analysis, statistical tests such as the chi-square test, one-way ANOVA, and Kruskall Wallis test (with Bonferroni post hoc test (if necessary)) are used. Regarding the main effects, linear mixed models will be applied for variables with normal distribution, considering the interaction between the factors time (before interventions, after 08 sessions, and 4-week follow-up) and groups. The data will be presented as mean, standard deviation, and the difference between means, and a 95% confidence interval of these differences. Sensitivity analysis will assess the missingness mechanism, and the data analysis approach will be the intention-to-treat method. All analyses will be done using SPSS version 26 (Chicago, IL, USA), and the significance level for all tests will be 5% [40].

Furthermore, Cohen *d* will be calculated to identify the effect size between the groups. The following interpretation of the Cohen *d* value will be used as a basis: around 0.2 (weak), around 0.5 (moderate), and above 0.8 (strong) [40].

## Discussion

The evaluation of the effects of applying PBMT on CNNP has evolved over the years. From the first pioneering studies to the most recent systematic reviews with meta-analysis, accumulated evidence suggests that PBMT in low-intensity form may be a promising, non-invasive therapeutic approach for relieving chronic pain and improving functionality in the cervical region. However, considering that the therapeutic approach is carried out in a multimodal way, it is necessary to understand the effects of combinations of therapeutic resources, mainly considering the plurality of conditions related to chronic pain.

This study was established to fill in the gaps in findings from previous studies [16–21]. Exercise and PBMT protocols are used clinically based on the needs of individuals with CNNP. Previous publications have demonstrated a great deal of miscellany in the composition of therapeutic exercises. Only immediate effects were often seen, and the exercises performed needed to meet the basic recommendations for improving variables related to individuals with CNNP. Focusing only on the intensity of the pain and not on the functional aspects [16–21]. This study differs from others by using clinically well-founded intervention protocols, functional variables, and longer follow-up times for the analyzed variables.

Therefore, this study fills a gap in the literature as it tries to verify the additional effects of a potentially practical resource associated with an intervention considered the first line of treatment. Based on previous evidence, this study’s findings will have the potential to positively impact clinicians, researchers, and individuals with CNNP by offering a treatment protocol tested with an appropriate methodology and consequently with due scientific support.

In the future, the findings of this study may directly impact self-care and self-management of therapeutic resources for individuals with CNNP. Expanding and favoring the understanding of your health condition and the most assertive non-medication and non-invasive clinical resources to be used for your health condition, especially about functionality. With interventions structured by well-designed studies and supported by solid methodological bases, the management of therapeutic resources becomes more assertive, favoring faster resolution of signs and symptoms attributed to CNNP and consequently reducing expenses for the health system.

### Supplementary Information

Supplementary Material 1. Description of the multimodal therapeutic intervention program, phase 1 and 2.

Supplementary Material 2.

## Data Availability

The datasets generated and/or analyzed during the current study are private due to our limited digital data stores for collective access. Still, they are available from the corresponding author on reasonable request.
