# Supplementary material for: Multimodal therapeutic intervention program associated with photobiomodulation therapy for individuals with chronic nonspecific neck pain: protocol for a clinical trial
Source: Trials. 2024 Jul 3;25:442. doi: 10.1186/s13063-024-08289-1 (PMC11221013; doi:10.1186/s13063-024-08289-1)
Supplement: Supplementary file 1 — Supplementary Material 1. Description of the multimodal therapeutic intervention program, phase 1 and 2. [file 13063_2024_8289_MOESM1_ESM.docx]

Table 1. Description of the multimodal therapeutic intervention programme, phase 1.

| Phase 1 | Intervention | Description | Duration |
| --- | --- | --- | --- |
| 1st to 4th Week | 1 | The research participant sits in a chair with the cervical region in a neutral position and performs flexion, extension, inclination and rotation movements of the cervical region, without load at the maximum possible amplitude. | Three sets of 5 repetitions for each movement mentioned. |
|  | 2 | Research participant in the supine position on the stretcher, with the cervical region resting on the stretcher. Researcher in charge positioned in front, with fingers in a "bridge" position under the occiput. The head is gently tilted using the radial deviation of the wrist. Then, during the tilt, traction is applied to the cervical region. | Three sustained sets of 10" to 15" of oscillation each. |
|  | 3 | Research participant, supine, on a stretcher, cervical region off the stretcher. The researcher in charge stands in front of the stretcher, places one hand on the occipital region while the other hand is placed under the mandible. The head was kept in a neutral position. The researcher then tractions the cervical region, leaning backwards using the weight of his own body. | Three sustained sets of 10" to 15" of oscillation each. |
|  | 4 | Research participant in dorsal decubitus position, on stretcher, with cervical region outside the stretcher. The researcher in charge stands in front of the stretcher and places his right hand on the occipital region while his left hand is placed under the mandible. The researcher tractions and rotates the cervical region to the left, then to the right, changing the position of the hands. | Three sustained sets of 10" to 15" of oscillation each. |
|  | 5 | Research participant in dorsal decubitus position, on stretcher, with cervical region outside the stretcher. The researcher in charge positions his right hand on the occipital region while his left hand is placed under the mandible. The researcher in charge tractions the cervical region with the right hand and consecutively slides the mandible antero-posteriorly with the left hand. | Three sustained sets of 10" to 15" of oscillation each. |
|  | 6 | Research participant in dorsal decubitus position, on the stretcher, with the cervical region outside the stretcher. The researcher in charge stands in front of the stretcher, places his thumbs under the mandible and makes a lateral slide to the right and left, keeping the head in line with the body to avoid flexion. | Three sustained sets of 10" to 15" of oscillation each. |
|  | 7 | Research participant in the right lateral decubitus position on the stretcher, with the cervical region resting on the stretcher, with the right arm supporting the head and the left arm in elbow flexion over the body. The researcher in charge, positioned laterally to the stretcher, places one hand on the base of the scapula and the other stabilises the participant's shoulder, performing superior/inferior sliding and rotation and retraction movements of the scapula using the movement of the body itself. This intervention should be carried out bilaterally. | Three sets with 30" of oscillation for each movement bilaterally. |
|  | 8 | Research participant in the supine position on the stretcher, with the cervical region in a neutral position, resting on the stretcher, arms extended in parallel. Researcher in charge in front of the stretcher. Participant will actively contract the deep muscles (flexors, extensors and rotators) of the cervical region, without moving the cervical spine, using eye movements to help perform these exercises. | Three sustained sets of 10" to 15" of oscillation each. |
|  | 9 | Research participant in dorsal decubitus, on a stretcher, with the cervical region in a neutral position resting on the stretcher, arms extended in parallel. Researcher in charge in front of the stretcher. The participant will perform isometric contractions of the flexor, inclinator and rotator muscles against the manual resistance of the researcher in charge. | Three sustained sets of 10" to 15" of oscillation each. |
|  | 10 | The research participant, in a prone position on the stretcher, arms flexed and hands resting in front of the chest, with the trunk flexed and the cervical region in a neutral position, will contract the extensors of the cervical spine against gravity. | Three sustained sets of 10" to 15" of oscillation each. |
|  | 11 | The research participant sits in a chair with the cervical region in a neutral position and performs flexion, extension, inclination and rotation movements of the cervical region, using an elastic band as far as possible, with the researcher supporting the elastic band and positioning himself according to the movement to be performed. | Three sustained sets of 10" to 15" of oscillation each. |

Table 2. Description of the multimodal therapeutic intervention programme, phase 2.

| Fhase 2 | Intervention | Description | | Duration | |
| --- | --- | --- | --- | --- | --- |
| 5th to 8th  Week | 1 | | The research participant, in the prone position on the mat, with arms extended parallel to the body and knees bent, stabilises the cervical spine, then anteriorises the head and returns with the chin tucked in. | | Three sets with 10-15 second holds or 8-12 repetitions, 120 seconds apart. |
|  | 3 | | The research participant, on all fours on the mat, maintains support and stabilisation of the cervical spine, anteriorises the head and returns with the chin tucked in. | | Three sets with 10-15 second holds or 8-12 repetitions, 120 seconds apart. |
|  | 4 | | Research participant on all fours on the mat, stabilising the cervical spine, then raising the upper limb to 90° alternately, keeping the chin tucked in. | | Three sets with 10-15 second holds or 8-12 repetitions, 120 seconds apart. |
|  | 5 | | Research participant on all fours on the mat, stabilising the cervical spine, then raising the upper limb up to 90° alternately, and the contralateral lower limb, keeping the chin tucked in. | | Three sets with 10-15 second holds or 8-12 repetitions, 120 seconds apart. |
|  | 6 | | The research participant stands, stabilises the cervical spine, then anteriorises the head and returns with the chin tucked in. | | Three sets with 10-15 second holds or 8-12 repetitions, 120 seconds apart. |
|  | 7 | Research participant standing, performing dynamic isometric exercise with elastic band, on occipital base, with shoulders and elbows in 90° position, performing elbow extension movement with resistance from elastic band, keeping chin tucked in. | | Three sets with 10-15 second holds or 8-12 repetitions, 120 seconds apart. | |
|  | 8 | Research participant standing, performing dynamic isometric exercise with an elastic band, at the base of the occiput, with shoulders and elbows in a 90° position, performing an elbow extension movement with the resistance of the elastic band on the right, keeping the chin tucked in. | | Three sets with 10-15 second holds or 8-12 repetitions, 120 seconds apart. | |
|  | 9 | Research participant standing, performing dynamic isometric exercise with an elastic band, at the base of the occiput, with shoulders and elbows in a 90° position, performing an elbow extension movement with the resistance of the elastic band on the left, keeping the chin tucked in. | | Three sets with 10-15 second holds or 8-12 repetitions, 120 seconds apart. | |
|  | 10 | The research participant stands and performs a dynamic isometric exercise with an elastic band, at the base of the occiput, crossed in front, with shoulders and elbows in a 90° position, performing an oblique elbow extension movement to the right and left, keeping the chin tucked in. | | Three sets with 10-15 second holds or 8-12 repetitions, 120 seconds apart. | |
|  | 11 | Research participant sitting on a therapeutic ball on a mat. Maintains support and stabilisation of the cervical spine, performs anteriorisation of the head and returns with chin tuck. | | Three sets with 10-15 second holds or 8-12 repetitions, 120 seconds apart. | |
|  | 12 | The research participant sits on a therapeutic ball, on a mat, stabilises the cervical spine, then raises the upper limb to 90° alternately, anteriorising the head and returning with the chin tucked in. | | Three sets with 10-15 second holds or 8-12 repetitions, 120 seconds apart. | |
|  | 13 | The research participant sits on a therapeutic ball on a mattress, stabilises the cervical spine, then raises the upper limb to 90° and the contralateral lower limb alternately, anteriorises the head and returns with the chin tucked in. | | Three sets with 10-15 second holds or 8-12 repetitions, 120 seconds apart. | |
|  | 14 | Research participant standing with occipital region supported by therapeutic ball on rigid surface. Maintains support and stabilisation of the cervical spine, performs head anteriorisation and returns with chin tuck. | | Three sets with 10-15 second holds or 8-12 repetitions, 120 seconds apart. | |
|  | 15 | Research participant standing with frontal region supported by therapeutic ball on rigid surface. Maintains support and stabilisation of the cervical spine, performs head anteriorisation and returns with chin tuck. | | Three sets with 10-15 second holds or 8-12 repetitions, 120 seconds apart. | |
|  | 16 | Research participant standing with occipital region resting on therapeutic ball under surface, performing bilateral shoulder abduction with resistance from elastic band attached to feet, keeping chin tucked in. | | Three sets with 10-15 second holds or 8-12 repetitions, 120 seconds apart. | |
|  | 17 | Participant standing with frontal region resting on therapeutic ball under rigid surface, performs bilateral shoulder abduction with resistance from elastic band attached to feet, keeping chin tucked in. | | Three sets with 10-15 second holds or 8-12 repetitions, 120 seconds apart. | |
|  | 18 | Participant standing, with stabilisation of the cervical region, performing a dynamic lifting movement of the upper limbs, with a therapeutic ball, associated with flexion of the lower limbs, keeping the chin tucked in. | | Three sets with 10-15 second holds or 8-12 repetitions, 120 seconds apart. | |
|  | 19 | Participant standing, with cervical stabilisation, performing functional dynamic exercise with scapular adduction and shoulder external rotation with resistance from elastic bands crossed over the palms of the hands. | | Three sets with 10-15 second holds or 8-12 repetitions, 120 seconds apart. | |
|  | 20 | Participant standing, one step forward, maintains cervical stabilisation and performs functional dynamic exercise with bilateral shoulder extension and scapular retraction with elastic band resistance. The researcher in charge will be in front of the participant, stabilising the elastic band. | | Three sets with 10-15 second holds or 8-12 repetitions, 120 seconds apart. | |
|  | 21 | Participant standing one step forward, maintains cervical stabilisation and performs functional dynamic exercise with bilateral shoulder elevation and scapular protraction with elastic band resistance. The researcher in charge will be behind the participant stabilising the elastic band. | | Three sets with 10-15 second holds or 8-12 repetitions, 120 seconds apart. | |
|  | 22 | Participant standing one step forward, maintaining cervical stabilisation, performs a functional dynamic exercise of shoulder abduction and bilateral elbow extension with scapular retraction, with resistance from an elastic band, crossed over the palms of the hands. | | Three sets with 10-15 second holds or 8-12 repetitions, 120 seconds apart. | |
|  | 23 | Participant standing with bipodal support, maintaining cervical stabilisation, performing a functional dynamic exercise of bilateral shoulder elevation, elbow extension, with resistance from an elastic band, crossed over the chest. | | Three sets with 10-15 second holds or 8-12 repetitions, 120 seconds apart. | |
|  | 24 | Participant standing with bipodal support, maintaining cervical stabilisation, performs a functional dynamic exercise of bilateral shoulder elevation, elbow extension and wrist pronation, with resistance from an elastic band, crossed over the chest. | | Three sets with 10-15 second holds or 8-12 repetitions, 120 seconds apart. | |
